# Optimizing Selection of the Reference Population for Genotype Imputation From Array to Sequence Variants

**DOI:** 10.3389/fgene.2019.00510

**Published:** 2019-05-31

**Authors:** Adrien M. Butty, Mehdi Sargolzaei, Filippo Miglior, Paul Stothard, Flavio S. Schenkel, Birgit Gredler-Grandl, Christine F. Baes

**Affiliations:** ^1^Centre for Genetic Improvement of Livestock, Department of Animal Biosciences, University of Guelph, Guelph, ON, Canada; ^2^Select Sires Inc., Plain City, OH, United States; ^3^Department of Agricultural, Food & Nutritional Science, University of Alberta, Edmonton, AB, Canada; ^4^Qualitas AG, Zug, Switzerland; ^5^Animal Breeding and Genomics Centre, Wageningen UR Livestock Research, Wageningen, Netherlands; ^6^Institute of Genetics, Vetsuisse Faculty, University of Bern, Bern, Switzerland

**Keywords:** dairy cattle, sequencing, imputation, haplotypes, accuracy, selection

## Abstract

Imputation of high-density genotypes to whole-genome sequences (WGS) is a cost-effective method to increase the density of available markers within a population. Imputed genotypes have been successfully used for genomic selection and discovery of variants associated with traits of interest for the population. To allow for the use of imputed genotypes for genomic analyses, accuracy of imputation must be high. Accuracy of imputation is influenced by multiple factors, such as size and composition of the reference group, and the allele frequency of variants included. Understanding the use of imputed WGSs prior to the generation of the reference population is important, as accurate imputation might be more focused, for instance, on common or on rare variants. The aim of this study was to present and evaluate new methods to select animals for sequencing relying on a previously genotyped population. The Genetic Diversity Index method optimizes the number of unique haplotypes in the future reference population, while the Highly Segregating Haplotype selection method targets haplotype alleles found throughout the majority of the population of interest. First the WGSs of a dairy cattle population were simulated. The simulated sequences mimicked the linkage disequilibrium level and the variants’ frequency distribution observed in currently available Holstein sequences. Then, reference populations of different sizes, in which animals were selected using both novel methods proposed here as well as two other methods presented in previous studies, were created. Finally, accuracies of imputation obtained with different reference populations were compared against each other. The novel methods were found to have overall accuracies of imputation of more than 0.85. Accuracies of imputation of rare variants reached values above 0.50. In conclusion, if imputed sequences are to be used for discovery of novel associations between variants and traits of interest in the population, animals carrying novel information should be selected and, consequently, the Genetic Diversity Index method proposed here may be used. If sequences are to be used to impute the overall genotyped population, a reference population consisting of common haplotypes carriers selected using the proposed Highly Segregating Haplotype method is recommended.

## Introduction

Globally, over 2.6 million cattle have been genotyped to date and the number of genotyped animals is expected to further grow in the coming years^1^. Dairy cattle genotyping is typically performed using genotype arrays of low or medium densities. Variants on genotype arrays are not selected randomly, rather they are evenly distributed over the whole genome and selected for their high level of segregation across multiple breeds (Boichard et al., 2012). Such a selection of variants has the advantage of enabling the application of the same array for multiple breeds, thus simplifying comparison between breeds. A disadvantage, however, is that they show an ascertainment bias, and variants with a low minor allele frequency (MAF) are underrepresented in genotype array data. The term “rare variants” henceforth refers to variants with a MAF lower than 0.05. Depending on the number of animals included and the alleles they carry, each genomic dataset contains its share of rare variants.

The lack of knowledge about rare variants hinders the discovery of quantitative trait loci (QTL) that, for example, appeared recently in a population through mutation (Fritz et al., 2013). Observed low MAF of variants can also be due to natural or artificial selection against an allele that has a negative impact on animal fitness or performance, thus indicating that a rare variant could be linked to a trait of interest or even a lethal malformation. An example of a rare variant associated with a disease can be found in a study by Drögemüller et al. (2009) in which a variant with a MAF of 0.03 is associated to arachnomelia (a calf malformation also called spider legs) in Brown Swiss cattle. Errors during genotyping or sequencing can also lead to wrongly identified variants with low MAF (Zhang et al., 2016).

Whole-genome sequencing can help provide better insight about rare variants (Daetwyler et al., 2014) but the costs of Next-Generation Sequencing technologies are still too high for mass sequencing of animals (Fraser et al., 2018). Imputation allows inference of whole-genome sequence (WGS) information for animals genotyped with various arrays based on complete WGS information of a reference population. The *in silico* creation of WGS from the readily available high number of genotypes enables a drastic increase in genotypic information for a large number of animals. High levels of imputation accuracy, however, are needed to allow use of the predicted genotypes for genomic evaluation or GWAS as demonstrated by Marchini and Howie (2010). The imputation from 50K to HD has been widely studied, and accurate HD genotypes are routinely imputed in dairy cattle genetic evaluation centers (e.g., Hozé et al., 2013; Ma et al., 2013; Pausch et al., 2013). Imputation to WGS variants, however, still needs to be improved. Accuracy of imputation is influenced by: (a) the size of the reference population; (b) the imputation method; (c) the relatedness between the reference and the target population; (d) the genotyping densities used, the difference in the number of variants and the linkage disequilibrium between SNP of both low- and high-density panels; (e) the MAF of the variants considered; and (f) the genetic diversity of the reference population. A thorough review of the factors influencing accuracy of imputation in livestock species was written by Calus et al. (2014). The selection of animals to include in reference populations influences many of these parameters and is thus of high importance. Druet et al. (2014) stated that as the MAF of variants becomes lower, the method used to select animals to be included in the reference population becomes more important.

The international dataset created under the scope of the 1,000 Bull Genomes Project (Daetwyler et al., 2014) is a possible reference set for imputation of cattle array genotypes to WGS. Up to Run 5 of this project, most animals sequenced were selected for their high genetic contribution to the population of their breed (Goddard and Hayes, 2009). These key ancestors carry most of the common variants for the populations they were selected from but lack information on rare variants. Pausch et al. (2017) showed that overall average imputation accuracy of array genotypes to the variant list from the 1,000 Bull Genomes Project was greater than 90%, but that the imputation accuracy of rare variants did not reach 70%. Low imputation accuracy of rare variants hinders the discovery of causal variants, not only for highly polygenic traits, but also for recent mutations that lead to malformations or loss of fitness (Li et al., 2011). Zhang et al. (2017) showed that the lack of accuracy in imputation of variants with low MAF also limits the success of genomic selection, particularly for health traits. Improved accuracy of WGS imputation will not only increase the probability of discovering causal variants for newly recognized diseases or malformations, but will also enable more precise categorization and selection of variants for routine genomic selection programs for traditional and novel traits.

Various methods have been proposed to select animals for sequencing, the first of which relied solely on pedigree information and targeted influential ancestors of the population of interest. Boichard et al. (1997) developed a method to identify animals that have the greatest genetic contribution to a population based on its pedigree information. This method was implemented and widely distributed using the software PEDIG (Boichard, 2002). The key ancestors method, developed thereafter, relied on the numerator relationship matrix of the genotyped population of interest and also aimed to maximize the proportion of genes of the population captured by the selected animals (Goddard and Hayes, 2009). As the number of genotyped animals increased, selection methods have been adapted to consider genomic information. Methods were proposed which emphasize selection of animals carrying common haplotypes. Druet et al. (2014) presented a method maximizing the number of haplotypes selected. The key contributors method presented by Neuditschko et al. (2017) defines animals as informative based on the genomic relationship matrix of the population and aims to select individuals within possible subpopulations. Another selection method developed by Gonen et al. (2017) involved the algorithm AlphaSeqOpt that not only selects individuals that, together, represent the maximum haplotype diversity of a population, but also suggests different sequencing coverages in situations where the sequencing costs are predetermined. An optimized version of AlphaSeqOpt was proposed by Ros-Freixedes et al. (2017), similarly considering situations where the sequencing costs were predetermined, but additionally targeting haplotypes instead of individuals. This method was shown to improve the phasing accuracy of the reference population it formed, even if it still maximizes the proportion of the total haplotypes included. In contrast to the previously described methods, which target representative animals of a population, the Inverse Weighted Selection Method (Bickhart et al., 2016) was developed to prioritize individuals for their higher genetic diversity at the haplotype level, classifying animals based on the rarity of their haplotypes. The Inverse Selection Methods was shown to allow sequencing of the maximum number of haplotypes with the fewest number of animals. In this study, two new selection methods are presented: the optimized Genetic Diversity Index (GDI), which targets animals carrying more rare haplotype alleles than the average individuals and the Selection of Highly Segregating Haplotype (HSH), which aims at selecting animals whose haplotypes are highly segregating, but not selected yet. The GDI method aims to improve the accuracy of imputation of rare variants through selection of animals that, together, carry the most different haplotypes, whereas the HSH should help to improve overall accuracy through selection of animals that carry the highest segregating haplotypes not previously sequenced.

The objectives of this study were: (1) to describe two innovative methods to select animals for sequencing from a population, and (2) to compare these methods to two previously described selection methods: the key ancestors method and the Inverse Weighted Selection method.

## Materials and Methods

**FIGURE 1:**
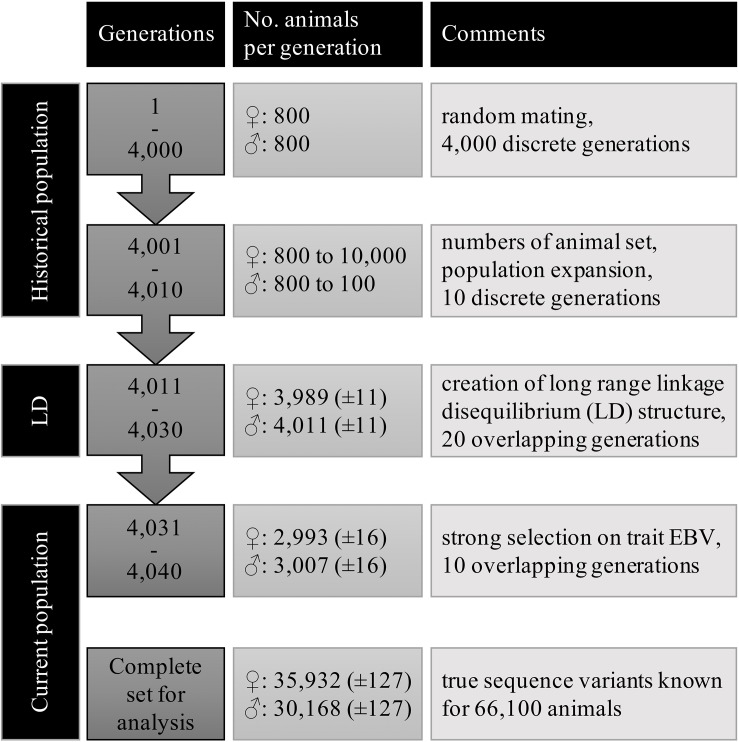
Structure and number of animals of the simulated populations.

Firstly, the WGS and high-density array genotypes of a dairy cattle population were simulated. Secondly, reference populations were created by selecting animals based on four different methods. Thirdly, a set of simulated target animals were imputed using the different reference populations. Finally, the imputation accuracies of the different methods were compared to each other considering sets of variants, defined depending on their MAF (Figure 1).

### Simulation

#### Population Structure

Large scale WGS data was simulated with the QMsim program (Sargolzaei and Schenkel, 2009) using three subsequent populations. First, a historical population was simulated to create linkage disequilibrium (LD) between the variants. Then, a second population, termed LongRangeLD, was simulated to increase long-range LD between variants. Finally, a third population (CurrentPop) was simulated for downstream analysis. CurrentPop simulated the latest years of dairy cattle breeding, in which few selected sires were used heavily in the breeding population.

The historical population considered an equal number of individuals from both sexes, discrete generations, random mating at the gametic level, no selection, and no migration. A total of 800 males and 800 females were simulated for 4,000 generations to achieve mutation-drift equilibrium. Ten further historical generations were generated expanding the population to 10,100 animals. In the last generation of the historical population, there were 100 males and 10,000 females.

The founders of LongRangeLD were all animals of the last generation in the historical population, after which each generation was composed of 8,000 animals. Through using different replacement rates, the 20 generations of this population overlapped. The total LongRangeLD population was composed of 168,100 animals. Founders of CurrentPop were 100 males and 4,000 females from the last generation of LongRangeLD and also 4,000 more females from the second-last generation of LongRangeLD. The 10 generations of this population had 6,000 animals and overlapped too. Finally, the complete population for downstream analysis had 66,100 animals, of which 30,168 (±127) were males. Migration was not simulated in any scenario. Further parameters used for both LongRangeLD and CurrentPop are presented in Table 1. The complete simulation process was replicated 10 times and the results reported are averages of the replicates.

**Table 1:** Parameters used for the simulation of the populations LongRangeLD and CurrentPop.

Parameter	LongRangeLD	CurrentPop
Number of generations	20	10
Litter size	1.0	1.0
Sire replacement rate	0.5	0.5
Dam replacement rate	0.3	0.3
Mating design	Positive assortative	Positive assortative
	on phenotypes	on EBV
Selection design	On phenotypes	On EBV
Culling design	Age	Low EBV
EBV estimation method	None	BLUP using the true additive
		genetic variance
Number of traits	1.0	1.0
Heritability	0.3	0.3
Phenotypic variance of trait	1.0	1.0


#### Genome

Gene-dropping simulation was completed using QMSim (Sargolzaei and Schenkel, 2009). The same genome was simulated for all populations. Cattle autosomal chromosomes were simulated with a length that followed the results presented by Bohmanova et al. (2010) and summed up to a total of 2,496 cM. Bi-allelic markers and QTL were randomly distributed over all chromosomes with equal MAF in the first historical generation. The QTL effects were sampled from a gamma distribution with a shape parameter of 0.4, following the results obtained by Hayes and Goddard (2001). The number of crossovers per chromosome was sampled from a Poisson distribution with mean equal to the chromosome length in centimorgans. The probability of a second crossover within 25 cM of a first recombination event was, therefore, lower depending on the proximity of crossovers. The mutation rate of the markers and the QTL was assumed to be 10^-4^. For each replicate, 8,622,767 markers and 4,000 QTL were generated.

#### Introduction of Genotyping Error and Selection of Variant Subsets

Selection of markers in the simulated data was performed to ensure that the MAF distribution followed that observed in the real data, described below. From all simulated variants, a first subset representing WGS was selected that contained all QTL. Then two subsets of the WGS were selected, which simulated high-density (HD) and medium density (50K) array genotype variant panels. In contrast to the WGS set, no QTL were allowed in the HD and 50K variant panels. Minor allele frequencies considered at this stage were computed considering a random sample of 30,000 animals from CurrentPop. Those animals represented 45% of the total population.

Real data comprised 425 Holstein (HOL) and 25 Red-Holstein animals from Run 5 of the 1,000 Bull Genomes Project (Daetwyler et al., 2014), 2,946 HOL animals (males and females) from the Canadian Dairy Network database (as of August 2017), and 36,157 HOL bulls with a North American identification tag born after 2010 for the WGS, HD, and 50K panels, respectively. The real WGS set was filtered for a minor allele count of 1 and was composed of 31,787,016 bi-allelic variants. Variants with a MAF lower than 0.1% were filtered out from the HD dataset. The real HD genotypes contained information for 587,817 bi-allelic variants. The same filter for variants with a MAF lower than 0.1% was applied to the 50K panel leading to 44,347 bi-allelic variants.

The number of selected variants per chromosome was proportional to the number of variants found in the real data. Variants were distributed by MAF in 50 bins. The sampling of the variants occurred randomly within the bin-by-chromosome groups with the function *sample()* in R, version 3.4.3 (R Core Team, 2017). The final simulated data was composed of 3,235,171 (±155,117), 571,661 (±6) and 44,288 (±0) variants for WGS, HD, and 50K, respectively. Genotyping error was introduced in the WGS based on error rates observed by Baes et al. (2014) using the HaplotypeCaller function of the Genome Analysis Toolkit with a multi-sample approach (McKenna et al., 2010; Table 2). Missing data was also added at this stage. Inclusion of genotyping errors and missing data in the genotypes was done using snp1101 (Sargolzaei, 2014).

**Table 2:** Rate of genotyping change as introduced in the simulated whole-genome sequence genotypes.

		Simulated genotypes including genotyping error and missing values			
		AA	AB	BB	-/-
**True genotypes**	**AA**	0.639	0.004	0.001	0.356
	**AB**	0.011	0.970	0.000	0.019
	**BB**	0.002	0.004	0.976	0.018


*As an example, 1.1% of the simulated AB genotypes were changed to AA genotypes. Values were retrieved from the study by Baes et al. (2014).*

#### Creation of the Reference Populations and the Validation Set

Groups of 50, 100, 200, 400, 800, and 1,200 animals were created from one pool of candidates using four selection methods. This pool of candidates was composed by all males of the CurrentPop and contained 30,027 (±108) bulls. As the 50K chip represents the preferred SNP chip for bull genotyping, animals were selected on their 50K haplotypes. The groups of selected bulls were later used as the reference populations for imputation from HD to WGS genotype density. Although imputation was done from HD to WGS genotype densities, selection of animals, when performed based on genotypes, was run on the 50K array panel to mimic again real situations, where the majority of the individuals would have only 50K genotype information. Haplotypes were defined as non-overlapping segments of 20 contiguous SNP of the 50K SNP panel throughout the study and had an average length of 1,082,875 bp (±264,426 bp). The same candidate pool was available for each method, so the same animal could be selected by multiple methods.

The selection methods were: (1) the key ancestors method, which used the additive genetic relationship matrix; (2) a combination of the newly developed Genetic Diversity Index and the simulated annealing algorithm (Kirkpatrick et al., 1983; Černý, 1985); (3) the Inverse Weighted Selection method (Bickhart et al., 2016); and (4) a second novel method aiming to select highly segregating haplotypes in the genotyped population that are not carried by any animal of the population of interest already sequenced. These methods are described in more details next. The 5,000 youngest animals (males and females) from CurrentPop that were not selected during the creation of the reference groups composed the target population of the imputation.

### Selection Methods

Selection of key ancestors was the method of choice to select the first animals sequenced in populations, as a representative genotyped group of animals from the population of interest was not needed (Daetwyler et al., 2014). This key ancestor method (AMAT) was chosen for comparison because of its frequent use and because it had indirectly a similar aim than the novel Selection of Highly Segregating Haplotype (HSH) method proposed here, i.e., selection of carriers of commonly found variants. Shortly after the first draft of the optimized Genetic Diversity Index (GDI) proposed here was designed, the paper of Bickhart et al. (2016) was published that presented the Inverse Selection Method (IWS). As GDI, this method aimed at selecting animals that are genetically more diverse in the pool of candidates. IWS seemed thus to be fairly comparable to GDI and was chosen to be included in this study. Other methods of animal selection for sequencing considered other objectives such as sequencing some animals at different coverages or combination of genotyping and sequencing, given a limited budget. In contrast, this study only considers situations where a given number of animals to sequence is given. Focusing on methods with similar aims than the novel methods proposed here seemed a way to allow for an in-depth analysis of them, as for example, differentiating accuracies of imputation of variants with different MAF.

#### Selection of Key Ancestors

The AMAT method aimed to identify animals explaining most of the genetic variation of a population following the equation $p_{n}\;=\;A_{n}^{-1}{}^{*}c_{n}$ where *p_n_* was a vector of the proportion of gene pool captured by the *n* selected animals, $A_{n}^{-1}$ was the inverse of the numerator relationship matrix of the *n* selected animals, and *c_n_* was a vector of the average relationships of the *n* selected animals with the entire population (Goddard and Hayes, 2009).

#### Inverse Selection Method

The IWS method developed by Bickhart et al. (2016) prioritized sequencing of rare haplotypes following the equation $Index\;=\;\sum_{i=1}^{NHAP}f_{i}^{2}-2f_{i}^{}+1$ where *NHAP* was the number of haplotypes and *f_i_* was the frequency of haplotype *i* in the population. This inverted parabolic function gave a high index value to individuals carrying haplotype alleles with low frequencies, as higher frequencies led to higher penalization (through the term-*2f_i_*). The computation of this index was iterative: (1) select the animal with the highest index; (2) recalculate the index of the remaining candidates without considering the haplotypes present in the genotypes of selected animals; and (3) pick out the next animal with the best new index. This method was used as it is implemented in the software program snp1101 (Sargolzaei, 2014).

#### Optimized Genetic Diversity Index

**FIGURE 2:**
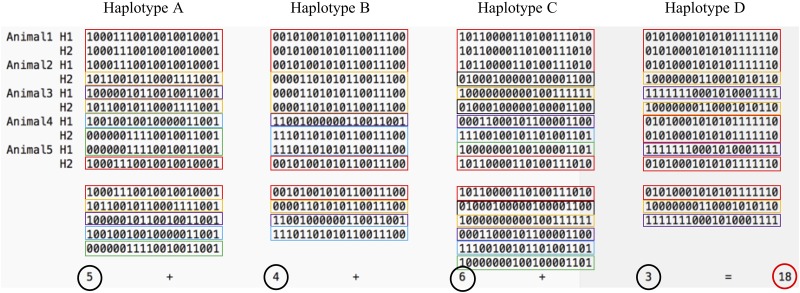
The Genetic Diversity Index is the sum of the unique haplotypes found in a group of animals. On this figure, five animals carry in total 18 unique haplotypes (5 variants of haplotypes A, 4 variants of haplotype B, 6 variants of haplotype C, and 3 variants of haplotype D). Colors highlight the unique haplotype alleles of each haplotype block.

Relying on a probabilistic optimization algorithm –simulated annealing (Kirkpatrick et al., 1983; Černý, 1985) – the proposed GDI method optimized the count of unique haplotypes of a group of animals composed of all previously sequenced animals and a defined number of sequencing candidates. The simulated annealing algorithm was developed to find the global optimum of a dataset with multiple local optima. The GDI of the whole group of animals was optimized with the simulated annealing algorithm permuting one candidate at a time and recalculating the index. The GDI was computed by summing the count of unique haplotype alleles present within a group of animals following the equation $Index=\sum_{i=1}^{NHAP}unique(HAP_{i})$, where *NHAP* was the number of haplotype blocks and *HAP_i_* were the haplotype variants in block *i*. Figure 2 gives an example of the index calculation based on five animals and four haplotypes. This method was also used as implemented in the program snp1101 (Sargolzaei, 2014).

#### Selection of Highly Segregating Haplotypes

To identify animals with the highest contribution to the population, the novel HSH method based on haplotype diversity was developed. The method had the following steps: (1) a haplotype library was created for all selection candidates using non-imputed genotypes. Haplotypes that appeared in less than 10 animals were discarded to reduce errors in the computation of their frequencies due to phasing error or haplotypes from other breeds; (2) contribution of each animal to the haplotype library based on the haplotypes’ frequency was calculated following the equation, $Index=\sum_{i=1}^{NHAP}f_{i}$, where *NHAP* was the number of haplotypes and *f_i_* was the frequency of haplotype *i* in the population. The animal with the highest Index value was then selected and; (3) frequencies of all haplotypes present in the selected animal were multiplied by a factor of 0.75 to penalize these already captured haplotypes. The factor for penalization is decided based on haplotypes frequency distribution in Holstein. Then the second most influential animal was selected based on highest contribution from the penalized haplotypes frequencies of all haplotypes it carries were multiplied again by the same factor of 0.75. After selecting an influential animal, total haplotype coverage (i.e., prevalence) was calculated for the new group of selected candidates. The process was repeated until the desired number of animals was selected, increasing the number of unique haplotypes selected with each animal, but avoiding selection of possible outliers (which carry many low-frequency haplotypes from another breed), for example from crossbred individuals as long as any non-outlier animals were still in the selection pool. Because the most frequent haplotypes were penalized first, the next animal chosen tended to carry haplotypes that are less frequent in the library or population. This method was also used as it is implemented in the software program snp1101 (Sargolzaei, 2014). The HSH method could accommodate any situation where some animals were previously sequenced, as the choice of the next influential animal is a function of already selected animals. Therefore, although the selected candidates may be different depending on which initial list of sequenced animals is used, the overall contribution to the population haplotypes should change only minimally.

### Measures of Diversity in the Reference Population

The level of genetic diversity was compared between reference populations. Next to the number of segregating variants as presented by Pluzhnikov and Donnelly (1996), the proportion of the total number of unique haplotypes alleles found in the candidate groups that were also found in the individuals selected for sequencing were used to compare the level of genetic diversity of the reference population of each scenario. The proportion of the rare haplotypes found in differently selected individuals was computed using the R package GHap (Utsunomiya et al., 2016). First, all haplotypes found within the candidates were identified. Second, the frequencies of the haplotypes within the candidates were computed. Finally, the proportions of haplotypes found in different groups were calculated. Following the construction of haplotypes when the animals were selected, haplotypes were built here again with 20-SNP windows and without overlap.

### Principal Component Analysis

Principal components analysis (PCA) is a statistical method that, when applied to genotypic data, allows detection of its structure (Ely et al., 2010). PCA was run on 50K genotypes of the candidate pool to determine the structure of the simulated population. This analysis was conducted using the implementation presented by Abraham and Inouye (2014) and available in snp1101 (Sargolzaei, 2014) with the following parameters: a maximum of 50 iterations were allowed, 40 principal components were computed and only variants with a MAF equal or higher than 0.01 were considered.

### Imputation

Following results presented by Whalen et al. (2018), the combination of the phasing software Eagle version 2.3.5 (Loh et al., 2016) and the imputation software Minimac3 (Das et al., 2016) – two programs developed for analysis of human data for which little to no family information is available – was used without pedigree information on the differently created reference populations to impute one set of target animals. Both software programs were used in their default mode. A linear genetic map of 1 cM per Mb was used to approximate the average recombination rate at phasing. From this step onward, all genotypes were reduced to the 10 first simulated chromosomes to reduce computation time and memory load. Imputed genotype calls only were used, not the genotype probabilities.

### Measure of Imputation Accuracy

**FIGURE 3:**
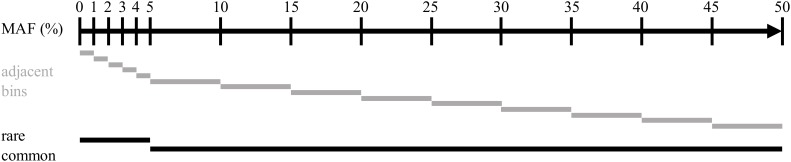
Representation of the distribution of the intervals of minor alleles frequencies used for assessing imputation accuracy.

Imputation accuracy was computed on multiple sets of variants for each scenario. Variants were distributed over multiple bins, depending on the MAF observed in the true genotypes of the target population of each simulation replicate. Two non-overlapping subsets containing common (MAF > 0.05) or rare (MAF = 0.05) variants were created, as well as a set of adjacent SNP bins. Variants were distributed following their MAF in the bins with boundaries at 0.00, 0.01, 0.02, 0.03, 0.04, 0.05, 0.10, 0.15, 0.20, 0.25, 0.30, 0.35, 0.40, 0.45, and 0.50. The bins were created to allow for the higher bound MAF to be included but not the lower bound. The composition of all bins is represented in Figure 3.

Imputation was evaluated at a per SNP basis by the squared correlation between the true and imputed genotypes and average. This accuracy measure, called allelic *R*^2^ by Browning and Browning (2009), is advantageous, as it is independent of the MAF of the variants imputed. Correlations between true and imputed genotypes were checked to ensure that negative correlations were not present so that no variants were filtered out at this stage. Accuracies of variants that were not segregating anymore after imputation were set to zero. Genotype concordance rates between all variants of the true and imputed genotypes were also computed on all variants. This measure represents the proportion of genotypes that are correctly imputed and allowed for evaluation of the imputation on a per animal basis.

### Performance of the Haplotype-Based Selection Methods With Crossbred Animals in the Candidate Pool

**FIGURE 4:**
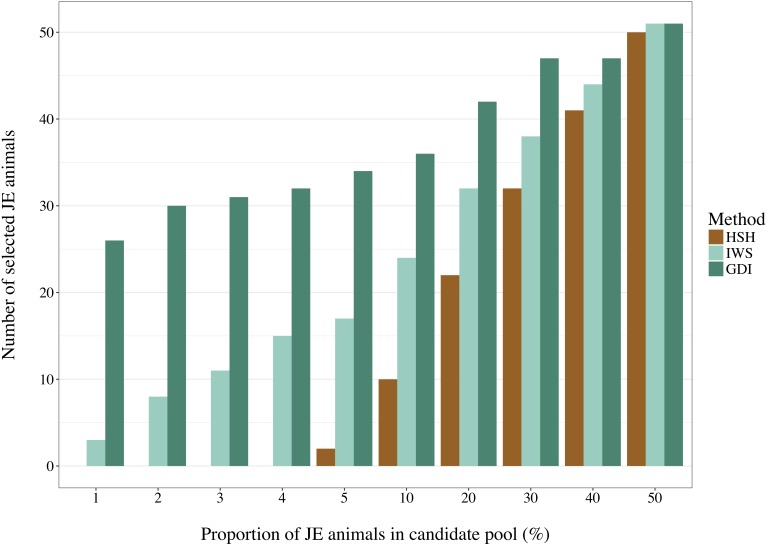
Number of Jersey (JE) animals selected by the Highly Segregating Haplotype selection (HSH), the Inverse Weighted Selection (IWS), and the Genetic Diversity Index (GDI) methods from candidate pools with different proportion of JE animals.

Selection of animals for sequencing is often run in one population at a time. Depending on the quality of the data recording, a proportion of the animals declared to be purely from one population may be crossbred or from another population. It is important that the method of selection avoids selecting individuals that are not part of the population of interest. BovineSNP50 genotypes of 16,420 Holstein and 2,920 Jersey (JE) males born after 2011 were retrieved from the Canadian Dairy Network database to create pools of 5,840 selection candidates with different degrees of admixture as presented on the horizontal axis of Figure 4. From the complete dataset, animals were randomly selected to enter each pool. The IWS, GDI and HSH methods were then used to select 100 animals out of each pool and the number of JE animals that were picked were counted.

### Statistical Tests of Average Differences Between Scenarios

After testing for the normality of the replicates within methods-by-reference size scenarios with Shapiro–Wilk tests, Kruskal–Wallis Rank Sum tests, and Wilcoxon Rank Sum statistical tests were performed for each MAF category to determine significant differences in accuracies among all methods or pairwise, respectively. The Bonferroni correction was used to adjust for multiple comparisons for an experimental-wise significance level of 0.05.

## Results

### LD Structure, MAF Distribution and Structure of the Simulated Population

**FIGURE 5:**
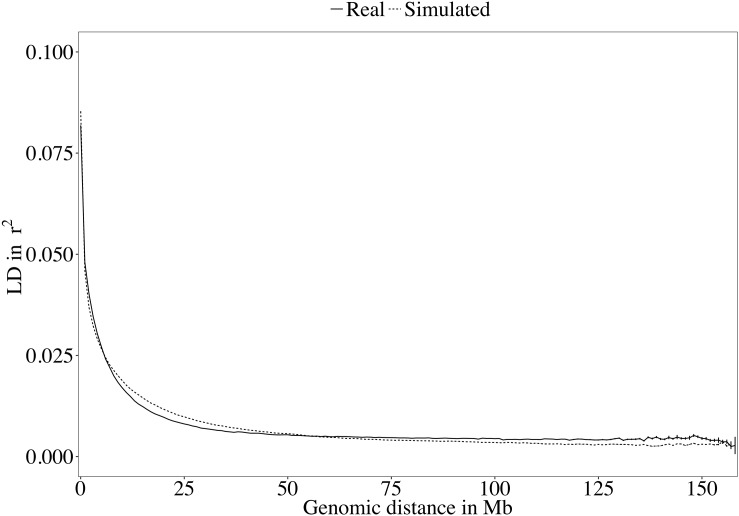
Decay of linkage disequilibrium (LD) over genomic distance in the real and the simulated sequences.

**FIGURE 6:**
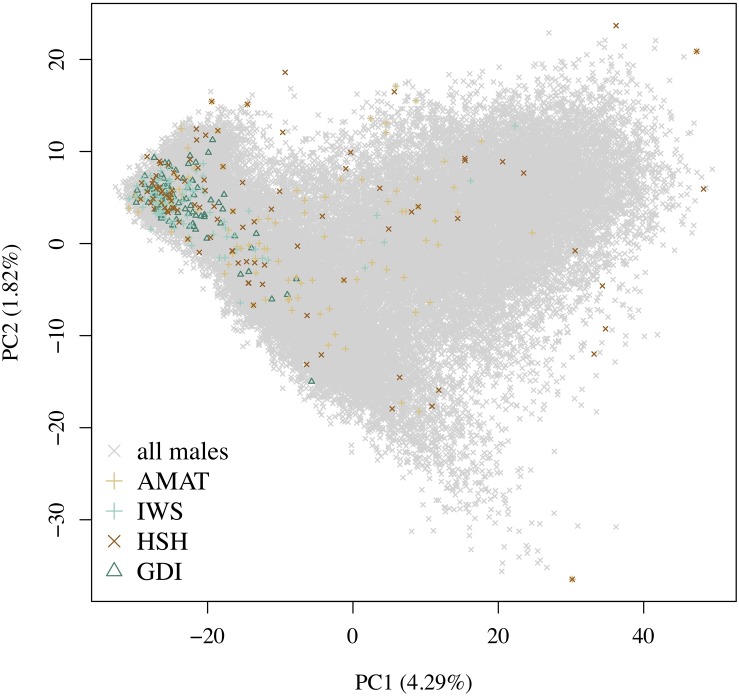
Distribution of the different groups of animals on the first and second principal components. The variance explained by the components is given in brackets. Gray crosses represent all the candidates, the green triangles are the animals selected by the key ancestors (AMAT) method, the purple plusses are the animals selected by the Inverse Weighted Selection (IWS) method, the green plusses are the animals selected by the Highly Segregating Haplotypes selection (HSH) method, and the blue crosses are the animals selected with the Genetic Diversity Index (GDI) method.

**FIGURE 7:**
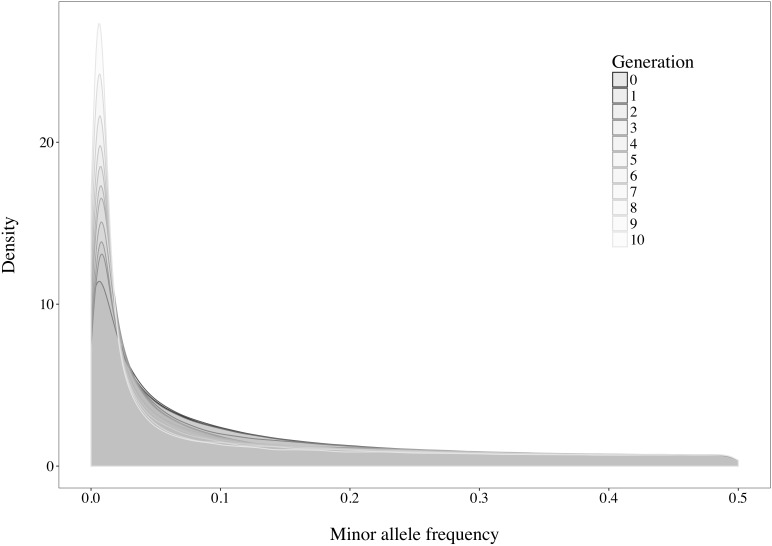
Density curves of the minor allele frequencies observed in the simulated population over the generations.

A rapid decrease in LD over increasing genomic distance was observed in both real and simulated genomic data (Figure 5). The high level of LD at distances shorter than 100kb in the real Holstein population already described by Sargolzaei et al. (2008) is mimicked in the simulation. Rare variants comprised 52.43% (±2.2%) of the WGS variants over the replicates. Principal component analysis showed a compactly distributed population on the two first components, which explained 6.11% of the total genomic variance (Figure 6). Spearman’s rank correlation between the first principal component and the generation of the animals was 0.87 (data not shown). Density curves of the MAF over the generations of the simulated population showed that an increasing number of variants became rare (Figure 7).

### Haplotype Coverage in the Reference Population

**FIGURE 8:**
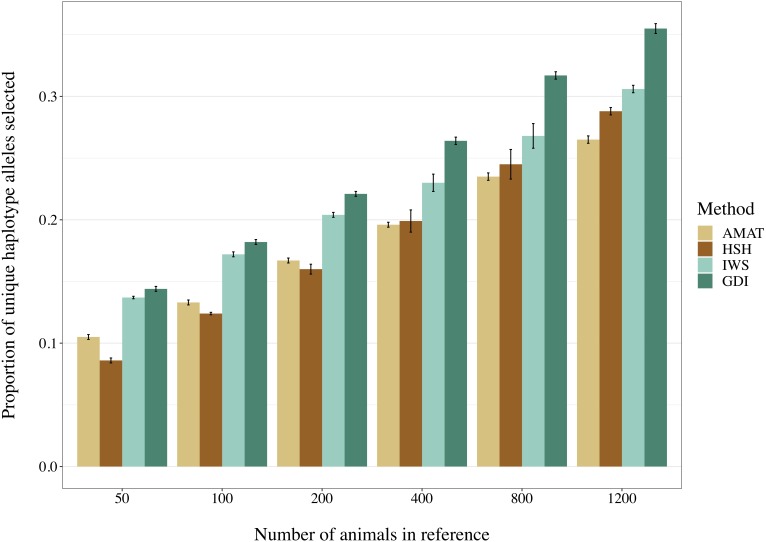
Selected proportion of unique haplotypes from the total haplotype library found in the reference group created with different selection methods. The methods compared are the key ancestors (AMAT), the Highly Segregating Haplotype selection (HSH), the Inverse Weighted Selection (IWS), and the Genetic Diversity Index (GDI).

The number of segregating variants and the proportion of unique haplotype alleles found in each reference population had a correlation of 0.68 (*P* < 0.0001). Increasing the number of animals in the reference population led to an increased proportion of unique haplotypes covered (Figure 8). Overall, haplotypes coverage ranged from 8.6% of the total haplotypes from the scenario with 50 animals selected on the basis of HSH, to 35.5% in the scenario including 1,200 animals selected through GDI. The reference groups created following the AMAT and HSH methods captured a lower proportion of the total haplotypes than reference populations created following the IWS and GDI methods. The proportion of haplotypes with a frequency equal or below 5% that were selected in each reference group followed the proportion of total haplotype selected.

### Overlap in Selection

The same pool of candidates was made available for selection for each method and reference size so that the same animals could be selected by multiple methods. The proportions of animals present in two groups for each reference size are shown in Table 3. Overlaps were higher between AMAT and HSH and between IWS and GDI. Small reference population sizes led to a higher proportion of animals found in multiple reference populations, with a maximum of 26% of animals found in common between the reference groups of AMAT and HSH that contained 50 animals in total. GDI did not have any overlap with AMAT or HSH for groups containing 50 and 100 animals. The overlap in the selected references of 100 individuals can be observed in Figure 6 where plusses, representing the animals selected with IWS, and crosses, representing the animals selected with GDI, are superposed.

**Table 3:** Proportion of animals overlapping between selection methods in reference populations of different sizes.

Size		Method		
		AMAT	HSH	IWS
**50**				
	HSH	0.26		
	IWS	0.04	0.00	
	GDI	0.00	0.00	0.08
**100**				
	HSH	0.23		
	IWS	0.03	0.01	
	GDI	0.00	0.00	0.1
**200**				
	HSH	0.20		
	IWS	0.05	0.03	
	GDI	0.01	0.02	0.14
**400**				
	HSH	0.13		
	IWS	0.04	0.06	
	GDI	0.02	0.03	0.09
**800**				
	HSH	0.09		
	IWS	0.03	0.12	
	GDI	0.03	0.06	0.12
**1,200**				
	HSH	0.08		
	IWS	0.03	0.16	
	GDI	0.04	0.09	0.12


*The methods were the key ancestors (AMAT), the selection of Highly Segregating Haplotypes (HSH), the Inverse Weighted Selection (IWS), and the Genetic Diversity Index (GDI).*

### Selection With Possible Crossbred Animals

With a pool composed of animals from two populations in a 50:50 ratio, no genotype-based method could avoid selecting at least half of them from the JE population (Figure 4). Differences were observed between methods in the more realistic scenarios with a proportion of 5% or less non-target animals. HSH did not select any JE animals until they comprised 5% of the candidate pool. In contrast, GDI already selected 58 JE animals when JE comprised 1% of the candidate pool. The 58 JE selected in this scenario were 45% of all JE animals present in the pool. In the scenarios with a candidate pool composed of 5% or less JE animals, IWS consistently selected only 5% of the JE animals.

### Accuracy of Imputation

Accuracies of imputation were observed on all variants and on two non-overlapping subsets: the rare variants with a MAF below 0.05 and the common variants with a MAF equal or above 0.05. Results are presented about these sets in the following order: first, all variants, then the rare variants and finally the common variants as the later showed re-ranking in comparison with the two other groups.

**FIGURE 9:**
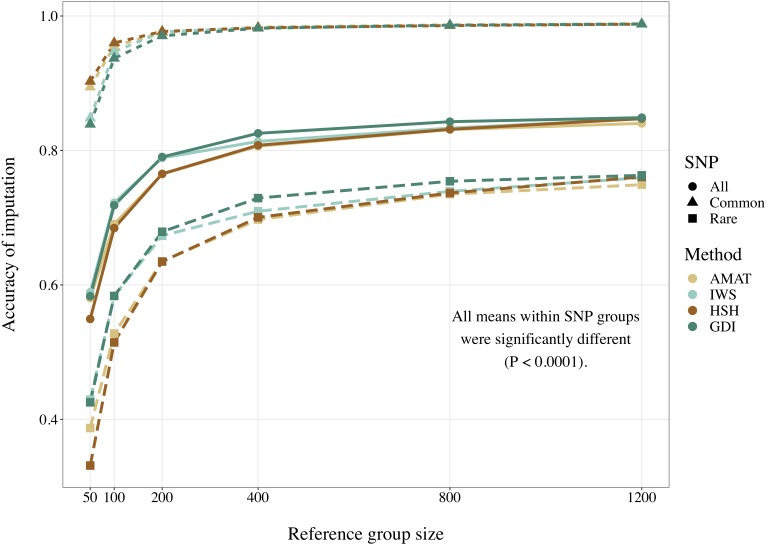
Accuracy of imputation for all SNP, only common SNP (minor allele frequency ≥ 0.05), or only rare SNP (minor allele frequency < 0.05), using reference population sizes from 50 to 1,200 individuals. The methods compared are the key ancestors (AMAT), the Highly Segregating Haplotype selection (HSH), the Inverse Weighted Selection (IWS), and the Genetic Diversity Index (GDI). All standard errors are below 0.013.

**FIGURE 10:**
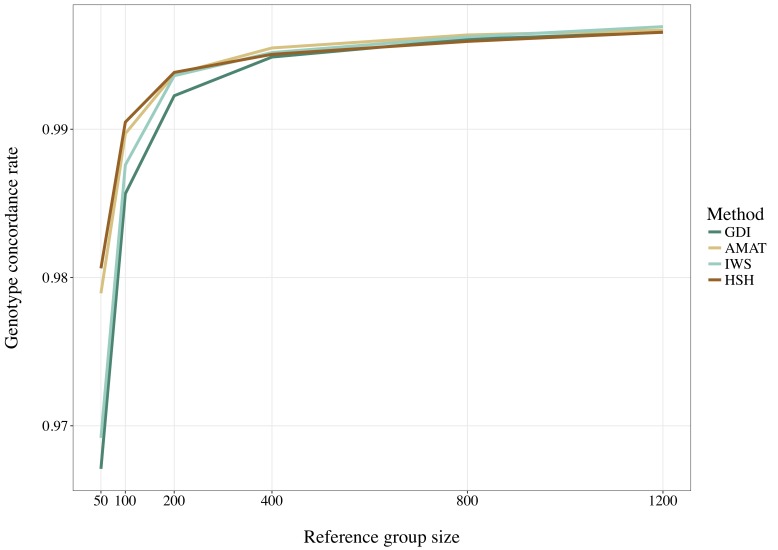
Genotype concordance rates for all SNP using reference population sizes from 50 to 1,200 individuals. The methods compared are the key ancestors (AMAT), the Highly Segregating Haplotype selection (HSH), the Inverse Weighted Selection (IWS), and the Genetic Diversity Index (GDI). All standard errors are below 0.009.

Considering all variants, accuracy of imputation reached values between 0.55 and 0.85, depending on the method used to create the reference groups and their sizes. Increasing the number of animals in the reference population led to corresponding increases in accuracies. Table 4 shows the accuracies of imputation reached in scenarios with 50, 200, and 1,200 reference animals selected by the four methods and across all adjacent MAF bins. In general, AMAT and HSH reached lower accuracies than IWS and GDI. The differences in accuracies, however, were smaller when the reference population size increased (Figure 9). In the scenario in which only 50 animals composed the reference population, IWS and GDI had the highest accuracies and were not significantly different (*P* > 0.05). AMAT had a significantly lower accuracy and the accuracy of HSH was even lower than that of AMAT (*P* < 0.0001) (Table 4). By increasing the size of the reference population to 100, 200, or 400 animals, differences in accuracy between AMAT and HSH were small, so that only two groups of methods, AMAT/HSH and IWS/GDI, could be differentiated. With reference groups of 800 and 1,200 individuals, only GDI and AMAT were significantly different (*P* < 0.0001), where GDI had the highest accuracy (0.944). Genotype concordance rates reached values above 0.96 in all cases (Figure 10). Significant differences between methods were only observed with reference populations comprising 50, 100, or 200 animals. Concordance rates were higher when animals were selected with HSH or AMAT than with IWS or GDI for reference sizes of 50 or 100 animals (*P* < 0.0001). Only the concordance rate of GDI for a reference population comprised of 200 animals was significantly lower than any other (*P* < 0.0001).

**Table 4:** Accuracies for reference populations of 50, 200, and 1,200 individuals and increasing MAF of the variants considered, all variants, the rare variants (MAF < 0.05), or the common variants (MAF ≥ 0.05).

	50				200				1,200			
MAF bin	AMAT	IWS	HSH	GDI	AMAT	IWS	HSH	GDI	AMAT	IWS	HSH	GDI
0.00-0.01	0.212^a^	0.282^b^	0.146^c^	0.281^b^	0.471^a^	0.527^b^	0.468^a^	0.540^b^	0.625^a^	0.641^a,b^	0.643^a,b^	0.647^b^
0.01-0.02	0.624	0.640	0.557	0.629	0.903	0.912	0.908	0.906	0.960	0.962	0.961	0.961
0.02-0.03	0.712^a^	0.704^b^	0.678^a^	0.691^b^	0.931	0.936	0.935	0.929	0.970	0.972	0.971	0.970
0.03-0.04	0.761^a^	0.732^b^	0.746^a^	0.725^b^	0.944^a,b^	0.948^a^	0.948^a,b^	0.940^b^	0.975	0.976	0.975	0.975
0.04-0.05	0.793^a^	0.754^b^	0.790^a^	0.745^b^	0.952	0.954	0.956	0.946	0.979	0.979	0.978	0.978
0.05-0.10	0.837^a^	0.786^b^	0.848^a^	0.776^b^	0.964	0.966	0.966	0.957	0.983	0.984	0.983	0.983
0.10-0.15	0.887	0.834	0.898	0.825	0.974	0.975	0.976	0.969	0.987	0.988	0.987	0.987
0.15-0.20	0.911^a^	0.865^b^	0.920^a^	0.855^b^	0.979	0.980	0.980	0.974	0.990	0.990	0.989	0.989
0.20-0.25	0.925^a^	0.878^b^	0.932^a^	0.870^b^	0.982	0.982	0.983	0.978	0.991	0.991	0.990	0.990
0.25-0.30	0.933^a^	0.892^b^	0.940^a^	0.881^b^	0.984^a,b^	0.984^a^	0.984^a,b^	0.980^b^	0.991	0.992	0.991	0.991
0.30-0.35	0.939^a^	0.904^b^	0.944^a^	0.896^b^	0.985^a,b^	0.985^a^	0.985^a,b^	0.982^b^	0.992	0.993	0.992	0.992
0.35-0.40	0.942^a^	0.908^b^	0.948^a^	0.900^b^	0.986	0.986	0.986	0.982	0.992	0.993	0.992	0.992
0.40-0.45	0.944^a^	0.911^b^	0.949^a^	0.904^b^	0.986	0.986	0.986	0.983	0.993	0.993	0.992	0.992
0.45-0.50	0.945^a,b^	0.914^b,c^	0.949^a^	0.908^b,c^	0.986^a^	0.986^b^	0.986^a^	0.983^b^	0.993^a^	0.993^b^	0.992^b^	0.993^b^
All	0.580^a^	0.589^b^	0.549^c^	0.583^b^	0.765^a^	0.789^b^	0.765^a^	0.790^b^	0.840^a^	0.847^a,b^	0.847^a,b^	0.849^b^
Common	0.894^a^	0.848^b^	0.903^c^	0.839^b^	0.976^a^	0.976^b^	0.977^a^	0.970^b^	0.988^a^	0.989^a,b^	0.988^b^	0.988^b^
Rare	0.387^a^	0.429^b^	0.331^c^	0.425^b^	0.635^a^	0.673^b^	0.635^a^	0.679^b^	0.749^a^	0.759^a,b^	0.761^b^	0.763^b^


*MAF bins “x-y” stands for “x < MAF ≤ y”. ^*a,b,c*^ Different letters represent significant differences in accuracies among the methods within a bin-by-size set of values (pairwise Wilcoxon Rank Sum Test significant with p-value after experimental-wise Bonferroni correction).*

When the accuracies of imputation were estimated on rare variants only, accuracies reached values between 0.33 and 0.76, but the rank of the methods from best to worst was consistent with results based on all variants (IWS/GDI > AMAT/HSH), and significant differences were also observed at any reference populations size (*P* < 0.0001). With reference size of 1,200 individuals, differences were only found between AMAT vs. HSH and AMAT vs. GDI, where AMAT had lower accuracy in both contrasts. In contrast, when only common variants were considered, the ranking was reversed: AMAT and HSH produced significantly higher accuracies than IWS and GDI (*P* < 0.0001). Accuracies took values as high as 0.99 and were never below 0.84. With a reference population of 50 animals, HSH reached a greater accuracy than AMAT and both were better than IWS and GDI. With reference sizes of 100 and 200, significant differences were again observed between the groups of methods AMAT/HSH and IWS/GDI (*P* < 0.0001). With 400 animals as reference, the accuracy reached by GDI was significantly lower than the other methods. Scenarios where 800 and 1,200 animals composed the reference population did not show difference in accuracy value before the fourth decimal. Although no change in the values was observed for these scenarios (Table 4), variances between replicates were very small (standard deviation < 0.004), therefore testing the methods against each other still led to significant results after correction for multiple testing.

**FIGURE 11:**
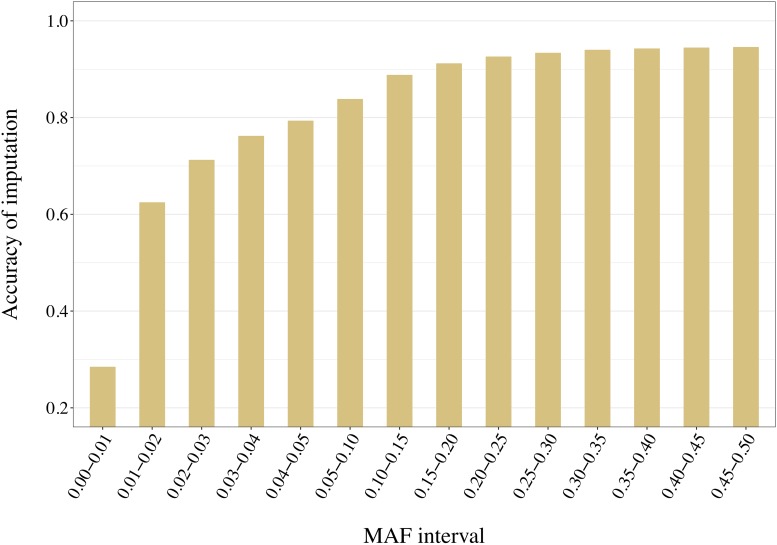
Accuracy of imputation increases with higher minor allele frequency (MAF) of the variants. Here the accuracies reached with 50 animals in a reference group of key ancestors (AMAT) are presented. MAF bins “x-y” stands for “x < MAF ≤ y”. All standard errors are below 0.008.

Distribution of the variants into 14 adjacent bins allowed a more precise evaluation of the effect of the reference composition on the imputation accuracy. With no exception, increased MAF led to increased accuracy values. For example, accuracies increased from 0.21 to 0.94 when using a reference group of 50 individuals selected with AMAT (Figure 11). Only pairs of contiguous MAF bins were analyzed and no significant differences within reference size-by-method scenario were found in imputation accuracy of variants with a MAF greater than 0.3 (*P* > 0.05).

## Discussion

In the first step of this study, the WGSs of a dairy cattle population were simulated. They were compared to currently available real Holstein sequence data to ensure they mimicked observed levels of LD and MAF distribution. In the second step, reference populations of different size were created with animals selected by both proposed novel methods as well as two other methods presented in previous studies. The selection methods were assessed with respect to their propensity to select animals that might not be of the population of interest, the genetic diversity of the groups of animals picked, and the distribution of those over generations. Finally, accuracies of imputation were compared for imputation runs with the different reference populations. The differentiation of imputation accuracy of variants with specific MAF allowed comparison between the strengths and weaknesses of each method of selection.

Different software programs were developed to simulate genomic information such as AlphaSim (Faux et al., 2016), ms2gs (Pérez-Enciso and Legarra, 2016), and QMsim (Sargolzaei and Schenkel, 2009). With its highly flexible genome and population configuration system, QMsim allowed for simulation of a great number of WGSs that had a LD structure properly following the parameters of the real data available. With the aim of simulating a Holstein population, only sequences of Holstein animals from the 1,000 Bull Genomes Project Run 5 were retrieved. These animals were mostly sequenced because they had a great genetic contribution to their population (Daetwyler et al., 2014). Although they are considered representative, these animals became influential as they were used heavily for breeding in their population and probably had a high genetic merit. They may, in fact, carry alleles at frequencies different from those in the overall population. This difference between the influential animals and the complete population limits the possible true closeness of the simulation with the whole real Holstein population. The LD level of the simulated sequences followed the real observed LD decay (Figure 5). Similarly, the distribution of the variants used in this work in MAF bins followed the distribution observed in real datasets.

Once the sequence was simulated, multiple reference populations were created by selecting animals using methods of selection that can be divided into two groups: AMAT and HSH, which mainly target animals that are carriers of commonly found haplotypes, whereas IWS and GDI are methods aiming to maximize the selection of animals carrying more haplotype alleles. Moreover, although AMAT keeps on searching for commonly found haplotypes, the penalization of those implemented in HSH leads to a shift from the search of commonly found haplotypes to rare ones. Through this shift, not only selection of common, but also of rare haplotypes is optimized. This shift, however, is highly dependent on the size of the candidate pool and the number of animals to be selected, as the increasing ratio of selected animals over the candidate pool facilitates the capture of more different haplotypes. A disadvantage of the haplotype-based selection method is that candidates must all be genotyped. In this sense, selection of animals for genotyping or sequencing in populations in which only a small proportion of individuals are genotyped should be done with AMAT, as long as a correct and complete pedigree is available.

Candidate pools for animal selection are often composed of individuals belonging to more than one population due to errors at the time of data recording, and thus crossbred animals could be erroneously selected. Testing methods for their tendency to pick crossbred animals revealed that methods targeting rare variants selected more animals that were not from the population of interest, which was expected. HSH was the only method in which no animal of the JE population was selected before their proportion in the candidate pool reached 5%, which can be considered a usual proportion of crossbred animals wrongly declared as purebreds (Figure 4). If GDI or IWS are used on real datasets, population structure analysis and analysis of the relationships between the candidates is essential to ensure that crossbred animals are removed prior to selection.

Following the control of the non-target animals selected with each method, a principal component analysis was used. This allowed for comparison of the distribution and overlap of the selected animals over the complete candidate pool. Methods targeting rare haplotypes picked the same animals more often (Table 3). The concentration of points representing the animals selected by IWS and GDI or the superposed dark and light brown points on Figure 6 follows the same idea. The animals selected for their higher genetic diversity were mostly of generation 1 and 2 out of the 10 simulated generations. Selection applied without allowing for migration in the simulated population led to a reduction of the MAF of the variants under selection pressure (Figure 7). Accordingly, the number of combinations of SNP alleles at the haplotype level was reduced, and less unique haplotypes alleles could be found in animals in generation three or more. Fewer unique haplotype alleles also led to higher haplotype frequencies of the remaining ones. Finally, carrying less unique haplotype and haplotypes alleles of higher frequencies, individuals of generation three or more were less likely to be selected by GDI and IWS.

It is of interest to assess the genetic diversity within and between the created reference populations. The proportion of selected haplotypes alleles increased with the number of animals selected, which was expected, as more animals can collectively carry additional different haplotypes. Similarly, when looking at the overlap of picked haplotypes alleles between methods, the methods presented more overlap if they targeted the common (AMAT, HSH) or the rare variants (IWS, GDI). Notably, when reference populations were smaller, animals selected with AMAT carried a greater number of different haplotypes than HSH. This can be explained by the following arguments. The HSH method makes sure that all commonly found haplotypes are selected before animals carrying rare variants get targeted, while AMAT relies solely on pedigree and thus has no possibility to consider the Mendelian sampling happening over generations. This limitation of AMAT, when compared to haplotype-based methods, was observed in our study when 1,200 animals comprised the reference group and only rare variants were considered. In this case, AMAT had a significantly lower accuracy than both HSH and GDI (Table 4). Moreover, it is likely that a real pedigree would contain errors that would not allow for a better haplotype coverage using AMAT than HSH as missing and incorrect information would impeach correct computation of the kinship among animals and thus the probable proportion of haplotypes they share. The pedigree-based method AMAT also showed a limitation once the number of animals increased, as redundancy of the added haplotype in the selected group was not directly avoided and effective Mendelian sampling could not be evaluated, which was in contrast to the results obtained for HSH. GDI consistently obtained greater haplotype coverage in the selected group of animals (Figure 8). This shows that the targeted optimization at the group level of the number of rare haplotypes was also achieved. Therefore, GDI and IWS seem to be the methods of choice when the objective is to select animals for their propensity to carry novel, rare or deleterious variants. The influence of the selection of genetically more diverse animals on the accuracy of selection, however, must be carefully assessed.

Overall, accuracies of imputation from HD to WGS were similar to previous results observed in real dairy cattle datasets (e.g., Pausch et al., 2017), although rare variants were kept throughout the whole analysis in the current study. Differences between scenarios were significant (*P* < 0.0001), however, the accuracies were mostly similar between methods. This was probably due to very low variance between the replicates, as the simulation algorithm is highly stable. All methods of selection avoided redundancy of the haplotypes selected, thus only minor differences between methods were observed after enough animals were selected. The greatest differences in accuracy of imputation between the methods were found when the reference population was small. Moreover, when observing genotype concordance rates, no differences were found when the reference populations comprised more than 200 animals. In contrary to the allelic *r*^2^ and as demonstrated in the review by Calus et al. (2014), the genotype concordance is dependent on the MAF of the variants considered and increases artificially with lower MAF. Differences in the distribution of the MAF of the rare variants between the reference population led to the observed re-ranking. Considering that animals selected with IWS and GDI were mainly from generation 1 and 2 of the simulated population (Figure 6), and that the MAF distribution of the rare variants shifted toward zero generation after generation (Figure 7), the MAF distribution within the rare variants category might be different between reference populations selected for high coverage of rare or common haplotypes. More different haplotype alleles were present in the reference populations selected with GDI and IWS (Figure 8), whereas animals selected with AMAT and HSH carried, as intended, more common variants. Animals selected with AMAT and HSH, however, still carried some rare variants but those had more often a MAF below 0.01. Figure 11 shows a distinctly bigger change in accuracy between monomorphic or rare variants with a MAF lower than 0.01 and rare variants with MAF between 0.01 and 0.05. It is this difference in the distribution of the MAF of the rare variants that explain the re-ranking of the methods between the genotype concordance and the allelic *r*^2^ values. Targeting rare haplotypes at selection (GDI and IWS) led to the creation of a reference population with more rare variants, but most of the added rare variants had a MAF between 0.01 and 0.05, whereas targeting common haplotypes led to the creation of a reference population carrying mainly common variants, but also some rare variants that mainly had a MAF below 0.01. Those variants with a MAF below 0.01 artificially increased the genotype concordance so that a re-ranking was observed.

Considering the re-ranking observed between method group, i.e., HSH/AMAT and IWS/GDI when looking at either rare or common variants, the method to select animals should be chosen using one of two principles: if the future imputed genotypes will be used as full genotypes and the imputation needs to be specially accurate for variants that will explain most of the genetic variation of a trait, animals should be selected using AMAT or HSH. In contrast, if future analysis will focus on the discovery of novel functional rare variants animals should be selected using IWS or GDI. Genotype concordance is the measure of imputation accuracy of choice when common variants that explain most of the genetic variance of most traits of interest for the dairy industry, are of interest for future analyses. Our results showed that genotype concordances with small reference populations were higher when the individuals were selected with AMAT or HSH. The first line of Table 4, where only the segregating variants with a MAF below 1% were considered, is a good example of the differences in accuracy of imputation for rare variants, variants that could have a novel deleterious effect. In this example, when the reference population only contained 50 animals, the difference in accuracy of imputation reached 0.18 points between the best (IWS) and the worst (HSH) methods. The accuracy of imputation increased with the MAF of the variants, but this increase stopped once segregation reached a level of 30% (Figure 11).

## Conclusion

Selection of animals for sequencing is an important task, as it greatly impacts the information gained about a population of interest, especially in populations with limited effective population size. Different selection methods are available that either rely solely on pedigree or that utilize information on previously genotyped individuals. In the first case, selecting key ancestors is highly recommended. Otherwise, the best method depends on the use of the future set of sequences. If the newly selected animals will be the first sequenced animals in their population and should allow for the overall imputation of the rest of the population, it is better to select animals carrying common haplotypes using the new HSH method instead of any of the other methods described in this study. If the resulting sequences of the selection of animals in a population will be used for discovery of new variants or should allow annotation of possible deleterious ones, animals carrying novel information should be selected and, consequently, the GDI method proposed here may be used.

## Author Contributions

AB, MS, and CB designed the simulation study and developed the new methods. FM, PS, and FS provided the data. AB, MS, and BG-G performed the data editing and the analysis. AB, MS, PS, and CB interpreted the results. AB wrote the manuscript. All authors read, commented on, and approved the final manuscript.

## Conflict of Interest Statement

MS was employed by HiggsGene Solutions Inc. and BG-G was employed by Qualitas AG. The remaining authors declare that the research was conducted in the absence of any commercial or financial relationships that could be construed as a potential conflict of interest. The reviewer MC declared a shared affiliation, with no collaboration, with one of the authors BG-G to the handling Editor at the time of review.

## Footnotes

1 https://queries.uscdcb.com/Genotype/cur_ctry.html, last accessed 2018-09-23
